# Mmu-miR-25-3p promotes macrophage autophagy by targeting DUSP10 to reduce mycobacteria survival

**DOI:** 10.3389/fcimb.2023.1120570

**Published:** 2023-05-15

**Authors:** Wenqi Yuan, Xuehua Zhan, Wei Liu, Rong Ma, Yueyong Zhou, Guangxian Xu, Zhaohui Ge

**Affiliations:** ^1^ Department of Orthopedics, General Hospital of Ningxia Medical University, Yinchuan, China; ^2^ Clinical Medicine School, Ningxia Medical University, Yinchuan, China; ^3^ The First Dongguan Affiliated Hospital, Guangdong Provincial Key Laboratory of Medical Molecular Diagnostics, School of Medical Technology, Guangdong Medical University, Dongguan, China

**Keywords:** mmu-miR-25-3p, tuberculosis, autophagy, DUSP10, macrophage

## Abstract

**Background:**

The present study aimed to investigate the regulation of miR-25-3p on macrophage autophagy and its effect on macrophage clearance of intracellular Mycobacterium bovis Bacillus Calmette-Guerin (BCG) retention based on the previous findings on the differential expression of exosomal miRNA in macrophages infected with BCG.

**Methods:**

Through enrichment analysis and Hub gene analysis, key differentially expressed miRNA and its target genes were selected. The targeted binding ability of the screened mmu-miR-25-3p and its predicted target gene DUSP10 was determined through the TargetScan database, and this was further verified by dual luciferase reporter gene assay. mmu-miR-25-3p mimics, mmu-miR-25-3p inhibitor, si-DUSP10, miR-NC,si-NC and PD98059 (ERK Inhibitor) were used to intervene macrophages Raw264.7. Rt-qPCR was used to detect the expression levels of mmu-miR-25-3p and DUSP10 mRNA. Western blot was used to detect the expression levels of DUSP10, LC3-II, p-ERK1/2, beclin1, Atg5 and Atg7. The autophagy flux of macrophage Raw264.7 in each group was observed by confocal laser microscopy, and the expression distribution of DUSP10 and the structure of autophagosomes were observed by transmission electron microscopy. Finally, the intracellular BCG load of macrophage Raw264.7 was evaluated by colony-forming unit (CFU) assay.

**Results:**

Bioinformatics analysis filtered and identified the differentially expressed exosomal miRNAs. As a result, mmu-miR-25-3p expression was significantly increased, and dual specificity phosphatase 10 (DUSP10) was predicted as its target gene that was predominantly involved in autophagy regulation. The dual luciferase reporter gene activity assay showed that mmu-miR-25-3p was targeted to the 3’-untranslated region (UTR) of DUSP10. The infection of BCG induced the upregulation of mmu-miR-25-3p and downregulation of DUSP10 in RAW264.7 cells, which further increased the expression of LC3-II and promoted autophagy. Upregulated mmu-miR-25-3p expression decreased the level of DUSP10 and enhanced the phosphorylation of ERK1/2, which in turn upregulated the expression of LC3-II, Atg5, Atg7, and Beclin1. Immuno-electron microscopy, transmission electron microscopy, and autophagic flux analysis further confirmed that the upregulation of mmu-miR-25-3p promotes the autophagy of macrophages after BCG infection. The CFU number indicated that upregulated mmu-miR-25-3p expression decreased the mycobacterial load and accelerated residual mycobacteria clearance.

**Conclusion:**

mmu-miR-25-3p promotes the phosphorylation of ERK1/2 by inhibiting the expression of DUSP10, thus enhancing the BCG-induced autophagy of macrophages. These phenomena reduce the bacterial load of intracellular Mycobacterium and facilitate the clearance of residual mycobacteria. mmu-miR-25-3p has great potential as a target for anti-tuberculosis immunotherapy and can be the optimal miRNA loaded into exosomal drug delivery system in future studies.

## 1 Introduction

Spinal tuberculosis is one of the major infectious diseases that seriously endanger human health. Hitherto, there has been no satisfactory treatment method. The bone marrow-blood barrier is altered in the sclerotic region surrounding tuberculous granulomas of spinal tuberculosis, characterized by structural and quantitative changes, trabecular dysplasia, partial closure of the medullary cavity, and the inclusion of the microscopic satellite tuberculosis lesions. Conventional formulations of first-line anti-tuberculosis drugs, such as isoniazid (INH), rifampicin (RFP), and pyrazinamide (PZA) cannot effectively penetrate the above tissue barrier and maintain effective bactericidal or bacteriostatic concentration in the lesion area due to their structural and pharmacokinetic characteristics. This in turn leads to residual chronic bacteria in tuberculosis granuloma in the diseased area and the drug resistance of tuberculosis bacteria, which is the primary reason for the ineffectiveness of conventional chemotherapy for spinal tuberculosis and the persistence and recurrence of the disease (Wang et al., 2007).

Exosomes consist of a class of nanovesicles that contain various substances derived from parental cells and transport them to recipient cells (Kalluri and LeBleu, 2020). Currently, exosomes are receiving increasing attention from various fields because they can be used as efficient shuttle vehicles to transport traditional medicines and also as genetic medicines (such as miRNA and proteins) with immunomodulatory functions (Salvi et al., 2018; Wu and Liu, 2018; Peng et al., 2020; Sharma and Johnson, 2020; Tang et al., 2020). Some studies on exosomes have focused on tissue repair and regenerative medicine, immune regulation, and nervous system diseases (Haney et al., 2015; Kalani et al., 2016; Lee et al., 2017; Izco et al., 2019). Nonetheless, the treatment of spinal tuberculosis with exosome-loaded anti-tuberculosis drugs and microRNAs (miRNAs) has not yet been studied. Therefore, we proposed to construct a novel anti-tuberculosis drug delivery system using exosomes as vectors loaded with RFP and specific miRNAs that have anti-tuberculosis and immunoregulatory functions. Based on the nanosized and low immunogenicity of exosomes, we suggested that conventional anti-tuberculosis drugs can cross multiple biological barriers *via* exosomes to effectively increase the effective drug concentration of spinal tuberculosis lesions. miRNA loaded with exosomes can exert an anti-tuberculosis immunomodulatory role and accelerate the clearance of macrophages to *Mycobacterium*. Therefore, identifying functional miRNAs is crucial for subsequent studies.

When *Mycobacterium tuberculosis* (MTB) infects the body, macrophages play a role in the first line of defense against infection (Cohen et al., 2018) and initiate multiple mechanisms, such as intracellular autophagy, to inhibit or eliminate MTB. Several studies have shown that miRNAs are involved in the regulation of autophagy and induce autophagy in macrophages to clear the intracellular MTB by regulating autophagy-related genes, the formation of autophagosomes in macrophages, and the maturation of autophagosomes (Deretic et al., 2006; Wang et al., 2013; Kim et al., 2015; Guo et al., 2017). In previous studies, we completed the extraction and identification of exosomes of the macrophages infected by *Mycobacterium bovis* Bacillus Calmette-Guerin (BCG). The miRNA was analyzed by high-throughput sequencing analysis of the differences in the exosomes of the macrophages after a 72-h BCG infection (Zhan et al., 2022). A total of 1853 miRNAs were detected, of which 506 overlapped between the BCG-infected and uninfected groups. The data showed that compared to the uninfected group, the expression of 20 miRNAs was upregulated, and that of 7 miRNAs was downregulated in the infected group (**Table 1**).

**Table 1:** Differentially expressed miRNAs and their expression levels (Zhan et al., 2022).

miRNAs	Fold Change	log2 FC	p-value	Exp level	Up/down
mmu-miR-27b-3p	1.17	0.22	2.22E-02	high	up
mmu-miR-93-5p	1.19	0.26	2.84E-02	high	up
mmu-miR-25-3p	1.33	0.41	3.98E-02	high	up
mmu-miR-1198-5p	1.16	0.21	4.19E-02	high	up
mmu-let-7c-5p	1.28	0.36	4.36E-02	high	up
mmu-let-7a-5p	1.28	0.36	4.36E-02	high	up
mmu-miR-7658-5p	inf	inf	1.44E-02	low	up
mmu-miR-7069-5p	inf	inf	2.85E-02	low	up
mmu-miR-8092	inf	inf	3.35E-02	low	up
mmu-miR-98-5p	1.18	0.24	1.10E-02	middle	up
mmu-miR-212-3p	1.38	0.47	1.36E-02	middle	up
mmu-miR-181b-5p	1.25	0.32	1.78E-02	middle	up
mmu-miR-3057-5p	1.40	0.49	2.00E-02	middle	up
mmu-miR-203-3p	2.55	1.35	2.23E-02	middle	up
mmu-miR-6516-5p	3.40	1.77	2.62E-02	middle	up
mmu-miR-181d-5p	1.97	0.97	3.38E-02	middle	up
mmu-miR-30a-3p	1.30	0.37	3.56E-02	middle	up
mmu-miR-1933-3p	2.03	1.02	3.77E-02	middle	up
mmu-miR-148b-5p	1.70	0.76	3.80E-02	middle	up
mmu-miR-99b-3p	1.34	0.42	4.88E-02	middle	up
mmu-mir-7018-p5	0.28	-1.85	2.38E-02	middle	down
mmu-miR-194-5p	0.69	-0.54	2.67E-02	middle	down
mmu-miR-301b-3p	0.82	-0.28	3.30E-02	middle	down
mmu-miR-5110	0.25	-2.02	3.61E-02	middle	down
mmu-miR-144-3p	0.28	-1.83	3.74E-02	middle	down
mmu-miR-874-3p	0.66	-0.59	4.05E-02	middle	down
mmu-miR-363-3p	0.34	-1.54	4.92E-02	middle	down

The mitogen-activated protein kinase (MAPK) signal transduction pathway is one of the major regulatory mechanisms in eukaryotic cells. Its signal transduction occurs through sequential phosphorylation of MAPKKK (mitogen-activated protein 3 kinase), MAPKK (mitogen-activated protein 2 kinase), and MAPK. MAPK is a highly conserved serine/threonine protein kinase that is part of a key signaling transduction system (Krishna and Narang, 2008). After activation by upstream kinases, different subfamilies regulate various physiological processes in cells, including autophagy, inflammation, stress, and cell growth, development, differentiation, and death (Bogoyevitch and Kobe, 2006; Park et al., 2014; Zhou et al., 2015). Since phosphorylation is required to activate the MAPK signaling pathway, dephosphorylation of the members of the DUSP (dual-specificity protein phosphatase) family plays a key role in controlling MAPK signaling. However, the function of DUSPs in autophagy is yet to be explored. Nomura et al. (Nomura et al., 2012) observed that DUSP10 suppresses the activation of ERK. Lu et al. (Lu et al., 2020) used Raw264.7 cells and confirmed that DUSP10 (namely MKP-5) promotes the transformation of macrophages from M1 phenotype to M2 phenotype, and as an inflammatory inhibitor, it participates in obesity-induced adipose tissue inflammation and PA-induced macrophage inflammation through ERK, P38, and JNK.

In the present study, we conducted a bioinformatics analysis and screened out mmu-miR-25-3p and its target gene *DUSP10*; these participated in the autophagy of macrophages. Therefore, we hypothesized that miR-25-3p regulates autophagy of macrophages after BCG infection through DUSP10/ERK pathway and further affects their ability to clear BCG retention in the cells.

## 2 Materials and methods

### 2.1 Tissue culture medium and reagents

Dulbecco’s modified Eagle’s medium (DMEM) and fetal bovine serum (FBS) were produced by Gibco and purchased from Thermo Fisher Scientific (Waltham, MA, USA). Complete *Mycobacterium* medium was purchased from Shanghai Gene-Optimal Science & Technology Co., Ltd (Shanghai, China).

Opti-MEM I reduced serum medium was produced by Gibco and purchased from Thermo Fisher Scientific. FuGENE^®^ HD Transfection Reagent was obtained from Promega (Beijing) Biotech Co., Ltd (Beijing, China). Antibodies against DUSP10 (# ab228987) and LC3-I/II (# ab128025) were purchased from Abcam (Cambridge, UK). Antibodies against Beclin1 (# D160120) and Atg5 (# D121650) were procured from Sangon Biotech (Shanghai) Co., Ltd (Shanghai, China). Antibodies against Atg7 (# AA820) and p-Erk1/2 (# AF5818) were purchased from Beyotime Biotech Inc. (Shanghai, China). Antibody against β-actin (# P60710) was purchased from Servicebio (Wuhan, China). Horseradish peroxidase (HRP)-labeled goat anti-rabbit IgG (# SSA004) was purchased from SinoBiological (Beijing, China). Anti-Rabbit IgG (whole molecule)-Gold (# G7402) was purchased from Sigma–Aldrich (Shanghai) Trading Co. Ltd (Shanghai, China). ERK1/2 signaling inhibitor PD98059 (# HY-12028) was procured from MCE (MedChem Express, Monmouth Junction, NJ, USA).

### 2.2 Cell and BCG cultures

ATCC^®^ SC6003™ standard was strictly observed. Raw264.7 cells (Gene-Optimal, Shanghai, China) were cultured with DMEM containing 10% FBS and 1% penicillin-streptomycin (Solarbio, China) at a low passage number at 37°C in a 5% CO_2_ atmosphere. The *Mycobacterium bovis* strain Bacillus Calmette-Guérin (BCG, St. Pasteur 1173P2 strain) was purchased from Shanghai Gene-Optimal Science & Technology Co., Ltd. Complete mycobacterium medium (Gene-Optimal) was used to culture the BCG bacilli at 37°C. A turbidimetric assay was used to detect the optical density (OD) of the bacteria in the logarithmic phase of growth on a microplate reader (Multiskan FC, Thermoe) at a wavelength of 600 nm. BCG bacilli were harvested at OD_600 = _0.6–0.8 by centrifugation for 10 min at 4500 rpm; the cell pellets were resuspended in DMEM without antibiotics for subsequent use.

### 2.3 Infection assay

Mycobacteria in suspension and the logarithmic growth phase were counted with an electronic McFarland turbidity meter, and the number of mycobacteria was estimated by measuring the McFarland (McF). The mycobacteria count corresponding to 1 MCF was approximately 3 × 10^8^ colony-forming units (CFU)/mL. According to the standards set for infection steps (Singh et al., 2015; Bettencourt et al., 2017), RAW264.7 cells were seeded into the plates overnight 1 day prior to infection. On the day of infection, the cells were rinsed with (BI, Israel) three times at room temperature. DMEM supplemented with 10% FBS but without antibiotics replaced the original culture medium, following which the cells were infected with BCG at a multiplicity of 10 (MOI = 10) and incubated at 37°C under a 5% CO_2_ atmosphere for 4 h. Subsequently, the medium was discarded, and phosphate-buffered saline (PBS) (37°C) was selected to gently wash the cells three times to the remove extracellular mycobacteria. DMEM supplemented with 10% FBS containing antibiotics was used for the culture. All the BCG culture and infection experiments were accomplished at Ningxia Key Laboratory of Pathogenic Microorganisms (biosafety level-3) in the General Hospital of Ningxia Medical University, China.

### 2.4 Bioinformatics analysis

In previous studies, we extracted and identified the exosomes of the macrophages infected by BCG. The miRNAs were analyzed by high-throughput sequencing analysis of the differences expressed in the exosomes of the macrophages after 72-h BCG infection (Zhan et al., 2022). The high-throughput sequencing data were deposited in the Genome Sequence Archive at the National Genomics Data Center, China National Center for Bioinformation/Beijing Institute of Genomics, Chinese Academy of Sciences (GSA: CRA006010). In present study, based on the differentially expressed miRNAs of the sequencing, enrichment analyses of their targeted genes were subsequently performed using GO and KEGG. Further visual analysis of the target genes related to cell growth and death was carried out using the Metascape database and the CytoHubba plug-in available in Cytoscape (v3.9.1).

### 2.5 Cell transfection

miR-25-3p mimics, miR-25-3p inhibitor, miR-NC (nontarget control oligonucleotide chain), three small interfering RNAs sequences against DUSP10 (**Table 2**), and their corresponding nontarget control siRNA (si-NC) were designed and generated by Shanghai Gene-Optimal Science & Technology Co., Ltd (**Table 2**). The confluency of RAW264.7 cells was 80% at the time of transfection. After cell digestion, the plates were prepared based on group classification. miR-25-3p mimics, miR-25-3p inhibitor, miR-NC, and all siRNAs were transfected into the cells using FuGENE^®^ HD transfection reagent as per the manufacturer’s instructions. 24 h after transfection, q-PCR was performed.

**Table 2:** Sequences of mimics and inhibitor of mmu-miR-25-3p and small interfering RNAs to *DUSP10* genes.

Gene name	Sequences (5’→3’)
miRNA nontarget control (miR-NC)	UUCUCCGAACGUGUCACGUTT
miR-25-3p-mimics	CAUUGCACUUGUCUCGGUCUGA AGACCGAGACAAGUGCAAUGUU
miR-25-3p-inhibitor	UCAGACCGAGACAAGUGCAAUG
siRNA nontarget control (si-NC)	UUCUCCGAACGUGUCACGUTT ACGUGACACGUUCGGAGAATT
si-DUSP10#1	GCUGCGAAUCUGACGUAUA UAUACGUCAGAUUCGCAGC
si-DUSP10#2	GGCCUUUCAUGGAGUACAA UUGUACUCCAUGAAAGGCC
si-DUSP10#3	CUAACCAGAUGGUCAACAA UUGUUGACCAUCUGGUUAG

### 2.6 Dual luciferase reporter gene assay

The wild-type (WT) 3’ UTR and a mutant type (MUT) sequence of DUSP10 were synthesized and cloned into a pmirGLO reporter vector. 293T cells were grown to 80% confluence, and the mixture containing the reporter vector and mmu-miR-25-3p mimics or inhibitors was cotransfected into different groups. A dual luciferase reporter gene assay kit (Beyotime, China, # RG089S) was selected to detect the relative luciferase activity.

### 2.7 Quantitative real-time PCR

RNA was extracted using an RNAeasy™ Animal RNA isolation kit with a spin column (Beyotime, #R0026), and miRNA was extracted using a SanPrep column miRNA extraction kit (Sangon Biotech, #B518811), according to the manufacturer’s instructions. RNA reverse transcription, cDNA verification, preparation of a real-time PCR system, and determination of the reaction conditions were carried out as described previously. *β-actin* and *U6* were the reference genes. The primers are listed in **Table 3**.

**Table 3:** Primer information.

Gene name	Primer Sequences (5’→3’)
*U6*	Forward primer: CTCGCTTCGGCAGCACA Reverse primer: AACGCTTCACGAATTTGCGT
*mmu-miR-25-3p*	Forward primer: GCGCATTGCACTTGTCTCG Reverse primer: AGTGCAGGGTCCGAGGTATT
*β-actin*	Forward primer: GCTTCTTTGCAGCTCCTTCG Reverse primer: GGCCTCGTCACCCACATAG
*DUSP10*	Forward primer: AGTAAATAGTCTGTGCGGGCT Reverse primer: GTTGTGCAGTCAGTTCCAGG

### 2.8 Western blotting assay

A membrane and Cytosol Protein Extraction Kit (Beyotime, #P0033) and Enhanced BCA Protein Assay Kit (Beyotime, # P0010S) were used for the extraction and concentration determination of total protein. Subsequently, the proteins were separated by SDS-PAGE and transferred to PVDF membrane (Aspen, China, #AS1021). Subsequently, the membranes were probed overnight at 4°C with primary antibodies to β-actin (1:1000), DUSP10 (1:1000), LC3-I/II (1:500), Atg5 (1:2000), Atg7 (1:1000), Beclin1 (1:2000), and p-Erk1/2 (1:1000). Then, the membranes were incubated with secondary antibody (HRP-labeled goat anti-rabbit IgG, 1:2000) for 30 min after washing with 1X TBST. Finally, the immunoreactive bands were visualized by chemiluminescence detection.

### 2.9 Immunoelectron microscopy

1×10^7^ Raw264.7 cells fixed with 2.5% glutaraldehyde at room temperature in the dark for 30 min, followed by three centrifugation washes at 3000 rpm, 4 °C for 10 min. The supernatant was discarded, and the pellet was resuspended in 0.1 M PBS (pH 7.4) and mixed with 2% molten agarose in the EP tube. Dehydration was performed with gradient ethanol at −20°C prior to embedding in LR white resin. Ultrathin sections (70–80 nm) were sliced, followed by immunolabeling. Then, the sections were blocked with 1% BSA/TBS blocking solution at room temperature for 30 min and incubated with the primary antibody (DUSP10, 1:1000) overnight at 4°C and secondary antibody (anti-Rabbit IgG (whole molecule)-Gold, 1:50) at room temperature for 20 min and then at 37°C for 1 h. Finally, the sections were stained with 2% uranyl acetate-saturated alcohol in the dark for 8 min and washed three times with 70% alcohol and ultrapure water. The images were captured using a Hitachi HT7800/HT7700 transmission electron microscope (Tokyo, Japan). Gold particles with a size of 10 nm in black indicate positive expression.

### 2.10 Autophagy flux analysis

RAW264.7 cells were seeded on 12-well plates and cultured overnight. The pAAV-mCherry-GFP-LC3B (Gene-Optimal, China) adenovirus was used to infect Raw 264.7 cells at an MOI of 100 for 24 h. Autophagic flux was determined by assessing the number of mCherry and GFP puncta under a NIKON Eclipse Ti confocal microscope.

### 2.11 Transmission electron microscopy

The cells were fixed, and sections were prepared, stained with uranyl acetate, and examined as described in 5.8 above, except that they were not stained with any antibodies. The autophagosomes were identified based on the morphological criteria described previously (Mizushima et al., 2010).

### 2.12 Colony-forming unit determination

Macrophages infected with BCG were used as controls. The other cells were transfected with mmu-miR-25-3p mimics, mmu-miR-25-3p inhibitor, and si-DUSP10 before BCG stimulation. The infection process was as described previously. DMEM-containing antibiotics were used to replace the medium, and the cells were cultured for 8 h. Subsequently, the lysed cells were serially diluted, coated with *Mycobacterium* solid culture medium (Gene-Optimal, China, #GOMY0020), and cultured at 37°C for 2-3 weeks; then, the number of colonies in each culture dish was counted.

### 2.13 Statistical analysis

SPSS Statistics 26 software (IBM Corporation, NY, USA) was utilized for data analysis. The measurement data were presented as mean ± standard error of the mean (SEM). Group comparison was tested using one-way analysis of variance (ANOVA). Significant results of ANOVA were further analyzed using the LSD (Least Significant Difference) test and Dunnett T3 test. Statistical comparison between the two groups was tested using Student’s t-test. p < 0.05 indicated a statistically significant difference.

## 3 Results

### 3.1 Enrichment analysis and hub genes

In a previous study, we extracted and identified the exosomes of the macrophages after BCG infection and analyzed the differentially expressed miRNAs in macrophage exosomes after 72-h infection by high-throughput sequencing (Zhan et al., 2022). The data showed that compared to the uninfected group, the expression of 20 miRNAs was upregulated and that of 7 miRNAs was downregulated in the infected group (**Table 1**). Next, we performed bioinformatics analysis on these 27 miRNAs.

**Figure 1:**
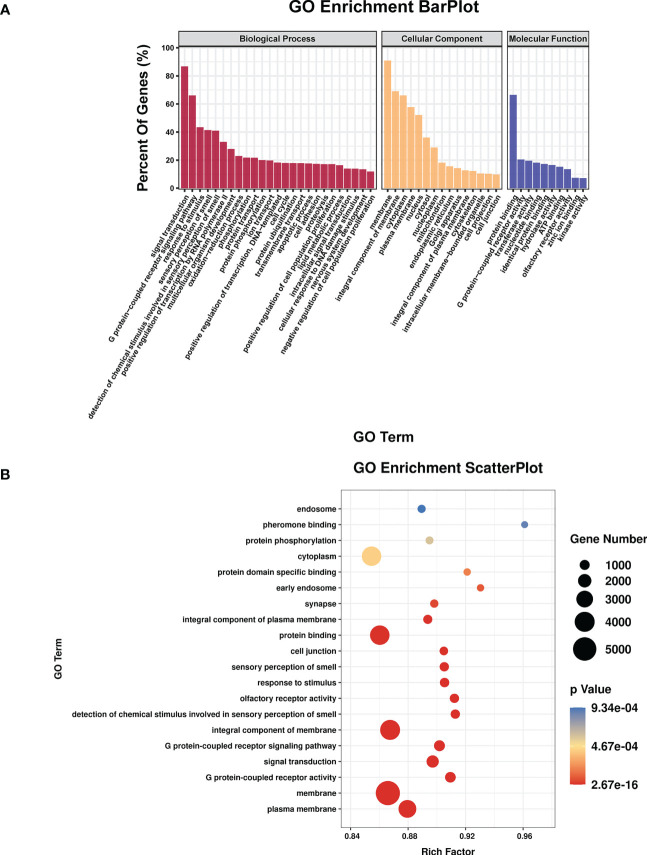
The results of GO enrichment analysis. **(A)** Bar plot shows GO terms of the biological process (BP), cellular component (CC), and molecular function (MF) categories. **(B)** Scatterplot shows the enrichment factors, indicates the ratio of the number of differentially expressed genes divided by the total number of genes annotated with a specific term. The smaller the value of p, the higher the degree of enrichment. The larger the diameter of a point, the greater the number of enriched genes.

**Figure 2:**
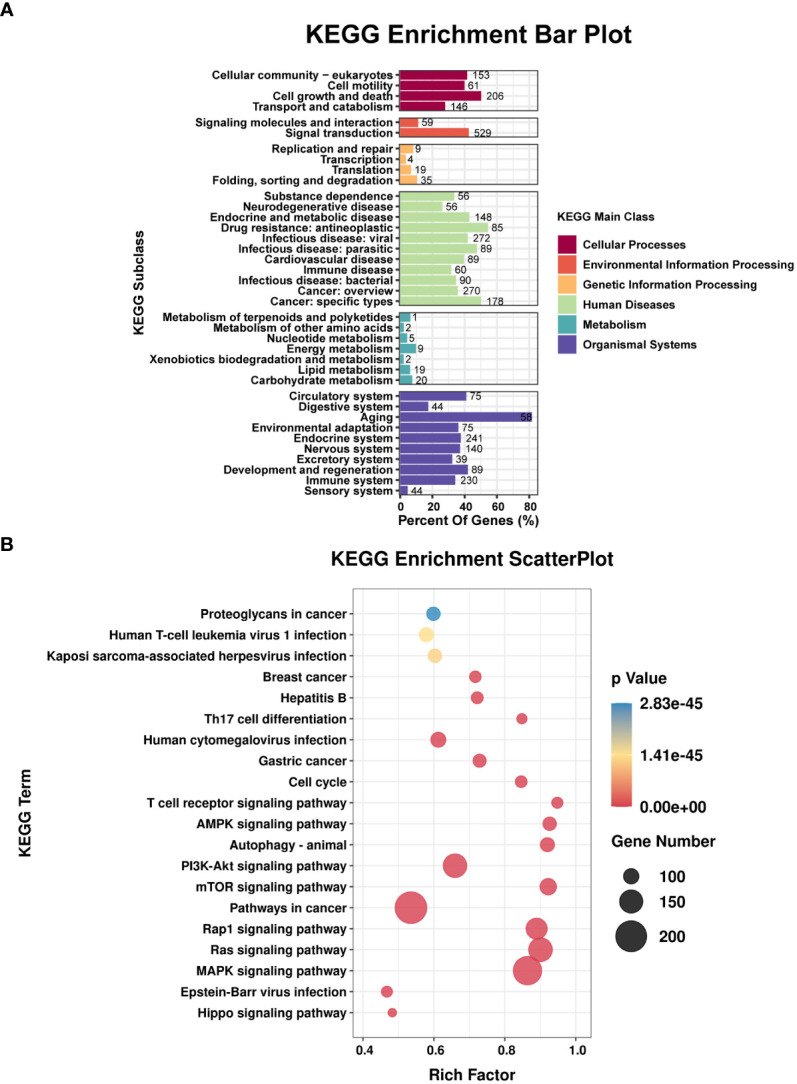
Results of the KEGG enrichment analysis. **(A)** Bar plot shows the KEGG subclasses of the target genes enriched in the six main KEGG classes. **(B)** Scatterplot shows the enrichment factors. The smaller the value of p, the higher the degree of enrichment. The larger the diameter of a point, the greater the number of enriched genes.

**Figure 3:**
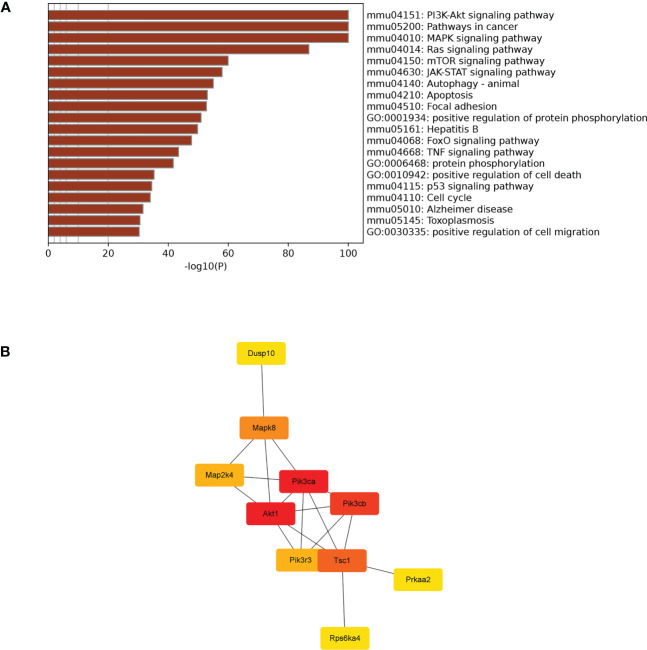
Visual analysis and hub genes. **(A)** Visualization of the 20 terms enriched with target genes related to cell growth and death as determined by Metascape. **(B)** Hub genes participating in apoptosis were identified by CytoHubba in Cytoscape (v3.9.1).

TargetScan (v5.0) and miRanda (v3.3a) were used to complete the Gene Ontology (GO) and genome pathway annotation. A total of 10667 target genes underwent enrichment analyses with OmicStudio, which is an online tool for bioinformatics. GO analysis indicated that these genes participated in multiple biological processes (**Figures 1A, B**). The bar chart analysis results of Kyoto Encyclopedia of Genes and Genomes (KEGG) enrichment analysis show that according to the number of enriched target genes, the top ten items are signal transduction, infectious disease: viral, cancer: overview, endocrine system, immune system, cell growth and death, cancer: specific types, cellular community – eukaryotes, endocrine and metabolic disease, and transport and catabolism (**Figure 2A**). It shows that these target genes play an important role in the regulation of cell growth, death, signal transduction, infection and immunity. The scatter plot analysis results of KEGG enrichment analysis show that according to the number of enriched target genes with significant differences, the top ten are pathways in cancer, MAPK signaling pathway, Ras signaling pathway, PI3K-Akt signaling pathway, Rap1 signaling pathway, mTOR signaling pathway, human cytomegalovirus infection, autophagy – animal, human T-cell leukemia virus 1 infection and AMPK signaling pathway (**Figure 2B**). Among them, Pathways in cancer has the largest number of target genes, but its rich factor is low, so it is not considered. The number of target genes enriched by MAPK signaling pathway ranks second, and its rich factor is greater than 0.8, while the rich factors of “autophagy-animal” and “mTOR signaling pathway” were the highest among the above 10 items (**Figure 2B**). Therefore, our subsequent analysis will focus on these three items. Next, we used Metascape to conduct in-depth data mining and online analysis of the functional visualization of these target genes. The results showed that autophagy, PI3K-Akt, mTOR, Ras, MAPK, and JAK-STAT were the top-ranked related signaling pathways (**Figure 3A**). Although the ranking of autophagy is not the top (**Figure 3A**), but some studies have proved that autophagy is closely related to the survival of MTB retained in macrophages (Gutierrez et al., 2004; Amano et al., 2006). Considering the above results comprehensively, we finally chose MAPK signaling pathway and autophagy as the direction of further analysis. Among these 27 miRNAs, 6 were highly expressed. Of these, mmu-miR-25-3p showed the highest fold-change (1.33) (**Table 1**), and hence was selected for further analysis of the Hubs gene of mmu-miR-25-3p. Current studies have confirmed that MAPK signaling pathway is closely related to autophagy regulation (Zhou et al., 2015; Wang et al., 2016). Therefore, we analyzed the target genes of mmu-miR-25-3p related to MAPK signaling pathway and autophagy through CytoHubba. Finally, *Dusp10*, *Akt1*, *Mapk8*, *Rps6ka4*, *Elk4*, *Map2k4*, *Tsc1*, *Pik3ca*, *Prkaa2*, *Pik3r3*, and *Pik3cb* were identified as Hub genes (**Figure 3B**). Since the function of DUSPs in autophagy has not been explored extensively, we chose to study the role of mmu-miR-25-3p and its target gene *DUSP10* in autophagy.

### 3.2 mmu-miR-25-3p targets the 3’-UTR of DUSP10

**Figure 4:**
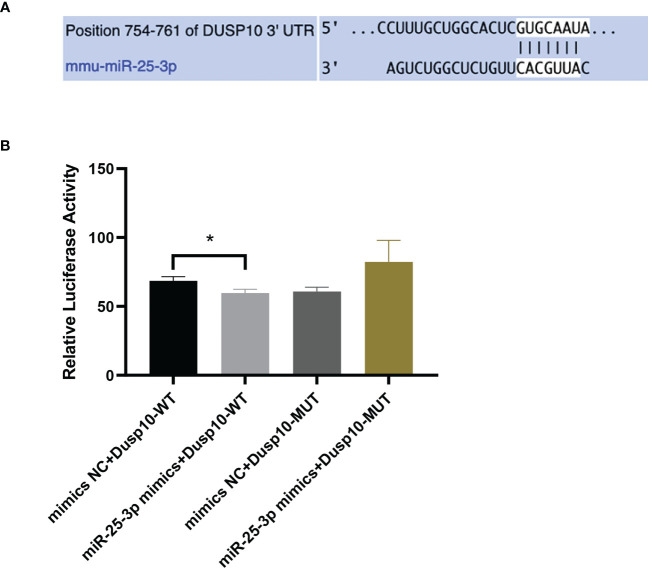
mmu-miR-25-3p targets the 3’-UTR of DUSP10. **(A)** Predicted sequential pairing of *DUSP10* seed and mmu-miR-25-3p 3’-UTR as determined by TargetScan (v8.0). **(B)** The dual luciferase gene reporter assay test results (This part of the experiment was repeated 5 times independently). *p < 0.05.

Bioinformatics analysis results predicted that *DUSP10* might be the target gene of mmu-miR-25-3p and that the seed sequence in mmu-miR-25-3p is complementary to the 754–761 oligomer in the 3’-UTR of DUSP10, as determined with TargetScan (v8.0) (**Figure 4A**). The base pairing type was an 8-mer, the Context++ score was -0.23, the Context++ score percentage was 92%, and the PCT value was 0.95. The fluorescence intensity of the miR-25-3p mimics + DUSP10 wild type (WT) 3’-UTR was obviously weakened compared to that of the NC mimics + DUSP10 WT 3’-UTR, and the difference between the groups was statistically significant (p < 0.05) (**Figure 4B**). The dual luciferase reporter gene activity assay confirmed that mmu-miR-25-3p targeted the 3’-UTR of DUSP10 and that DUSP10 was one of the target genes of mmu-miR-25-3p.

### 3.3 BCG infection induces alters mmu-miR-25-3p and DUSP10 expression in RAW264.7 cells

**Figure 5:**
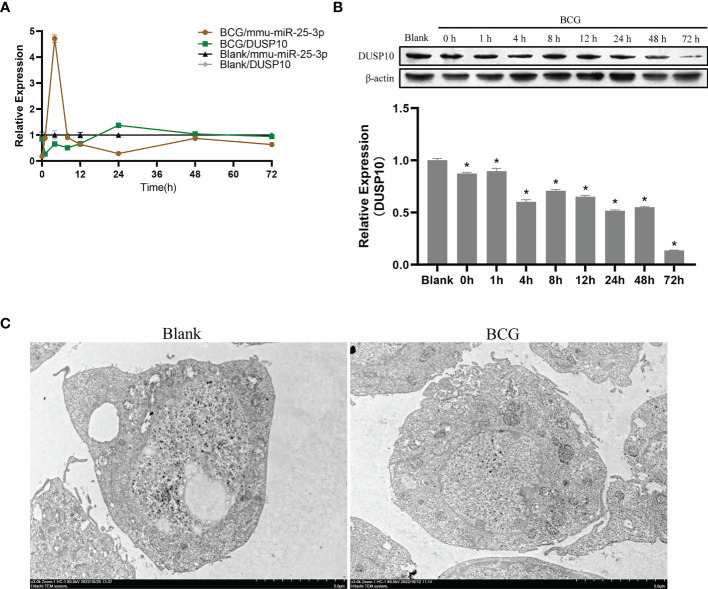
Infection of BCG alters the mmu-miR-25-3p and DUSP10 expression in RAW264.7 cells. **(A)** Expression trends of mmu-miR-25-3p and *DUSP10* mRNA in RAW264.7 cells. **(B)** Expression trends of DUSP10 in RAW264.7 cells. **(C)** After 8 h post-BCG infection at MOI 10, the localization of DUSP10 was observed by immunoelectron microscopy. All the experiments were repeated 3 times independently. Gold particles with a size of 10 nm in black indicate DUSP10 expression. Bars in D: 5 μm. *p < 0.05.

In innate immune cells, protein phosphorylation cascades regulate the activation of signaling pathways that are crucial for defense against bacterial infection (Zhang and Dong, 2005). Herein, we infected Raw264.7 macrophages with BCG at MOI 10 and detected the expression levels of mmu-miR-25-3p, *DUSP10* mRNA, and DUSP10 protein by qRT-PCR and Western blotting at 0, 1, 4, 8, 12, 24, 48, and 72 h post-infection. Compared to the blank group, the expression trend of mmu-miR-25-3p showed a peak expression at 4 h after BCG infection (p < 0.05), while the expression trend of *DUSP10* mRNA was significantly downregulated between 1 and 12 h post-infection (p < 0.05) (**Figure 5A**). Compared to the blank group, the expression level of DUSP10 protein was continuously downregulated after infection (p < 0.05) (**Figure 5B**). Immunoelectron microscopy detected a decreased abundance of DUSP10 in cells infected with BCG and DUSP10 proteins that were mainly localized in cell nucleus (**Figure 5C**). These results indicated an increased expression of mmu-miR-25-3p and a decreased expression of DUSP10 in RAW264.7 cells in response to BCG infection.

### 3.4 BCG infection alters the autophagy in RAW264.7 cells

**Figure 6:**
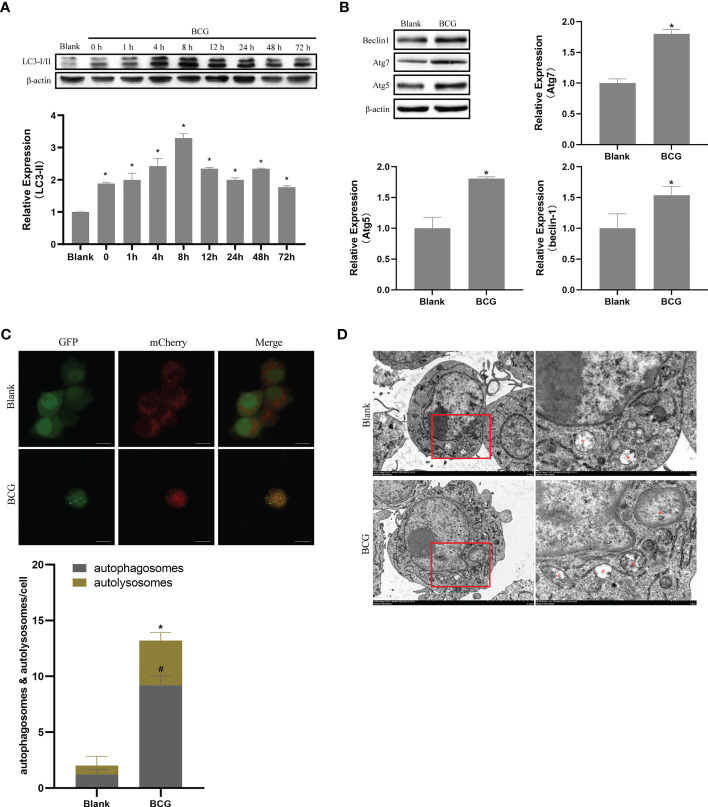
BCG infection alters autophagy in RAW264.7 cells. **(A)** Expression trends of LC-3I/II in RAW264.7 cells, *p < 0.05. **(B)** Levels of Atg5, Atg7, and Beclin1 at 8 h post-BCG infection, *p < 0.05. **(C)** RAW264.7 cells were infected with pAAV-mCherry-GFP-LC3B adenovirus (MOI = 100) for 24 h prior to 8 h of BCG infection (MOI = 10); mCherry-LC3B and GFP-LC3B puncta were observed by confocal microscopy, #p < 0.05 (compared with autophagosomes of Blank), *p < 0.05 (compared with autolysosomes of Blank). **(D)** After 8 h of infection at MOI 10, autophagic vesicles (marked with red *) were observed by transmission electron microscopy. All the experiments were repeated 3 times independently. Bars in C: 10 μm. Bars in D: 5 μm (×3.0k), 1μm (×8.0k).

Autophagy plays a critical role in the process of macrophage resistance against tuberculosis (Gutierrez et al., 2004; Amano et al., 2006). In the current study, Raw264.7 macrophages were infected with BCG at MOI 10, and the expression levels of LC3-II were detected at 0, 1, 4, 8, 12, 24, 48, and 72 h after infection. Compared to the blank group, the expression level of LC3-II increased after infection and peaked at 8 h post-infection (p < 0.05) (**Figure 6A**). Therefore, we further detected the expression levels of Beclin1, Atg5, and Atg7 at 8 h after infection, and found that the levels of these three autophagy-related proteins in BCG-infected macrophages were higher than those in the blank group (p < 0.05) (**Figure 6B**). The BCG-induced autophagy was further corroborated by ascertaining autophagy flux in cells expressing pAAV-mCherry-GFP-LC3B transduced by adenovirus. Indeed, accumulated autophagosomes (yellow puncta in Merge) and autolysosomes (mCherry-LC3B puncta), indicatives of the occurrence of autophagy were observed in the cytoplasm of BCG-infected Raw264.7 macrophages relative to the uninfected Raw264.7 macrophages (p < 0.05) (**Figure 6C**). Transmission electron microscopy (TEM) further confirmed that the number of autophagosomes in Raw264.7 cells at 8 h after BCG infection was significantly higher than that in the blank group (**Figure 6D**).

### 3.5 mmu-miR-25-3p/DUSP10 mediates autophagy upon BCG infection by regulating ERK1/2 phosphorylation

**Figure 7:**
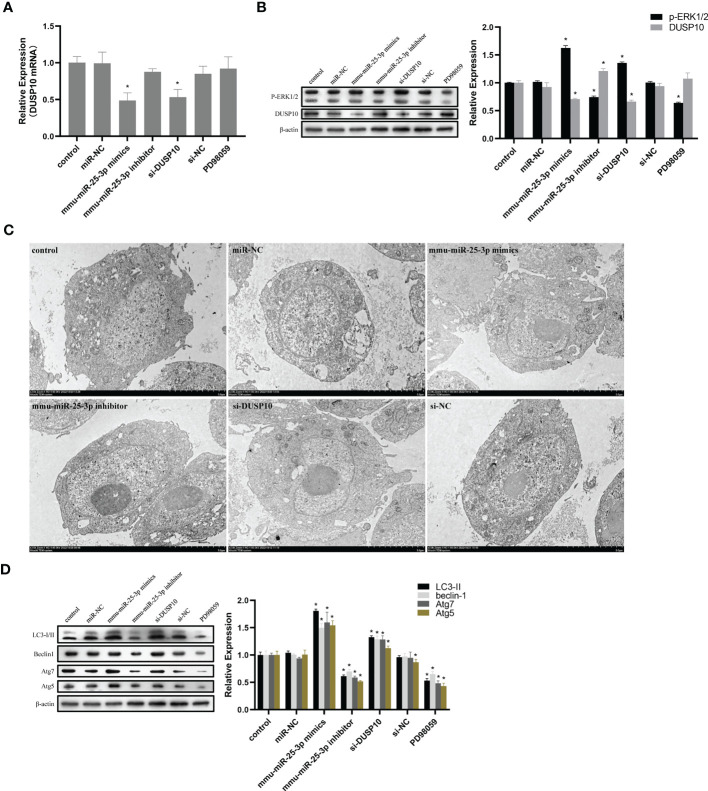
mmu-miR-25-3p/DUSP10 mediates autophagy upon BCG infection by regulating ERK1/2 phosphorylation. **(A)** 8 h post-BCG infection, the expression of *DUSP10* mRNA in the control group (only infected with BCG, no other interventions) and each intervention group (miR-NC, mmu-miR-25-3p mimics, mmu-miR-25-3p inhibitor, si-DUSP10, si-NC, and PD98059). **(B)** 8 h post-BCG infection, DUSP10 and p-ERK1/2 levels in the control group and each intervention group (miR-NC, mmu-miR-25-3p mimics, mmu-miR-25-3p inhibitor, si-DUSP10, si-NC, and PD98059) were analyzed by Western blotting. **(C)** After 8 h post-BCG infection at MOI 10, the localization of DUSP10 was observed using immunoelectron microscopy in the control group and each intervention group (miR-NC, mmu-miR-25-3p mimics, mmu-miR-25-3p inhibitor, si-DUSP10, and si-NC). 10-nm gold particles in black indicate DUSP10 expression. **(D)** 8-h post-BCG infection, levels of Atg5, Atg7, Beclin1, and LC3-I/II proteins were analyzed by Western blot in the control group and each intervention group (miR-NC, mmu-miR-25-3p mimics, mmu-miR-25-3p inhibitor, si-DUSP10, si-NC, and PD98059). All the experiments were repeated 3 times independently. Bars in C: 5 μm. *p < 0.05.

**Figure 8:**
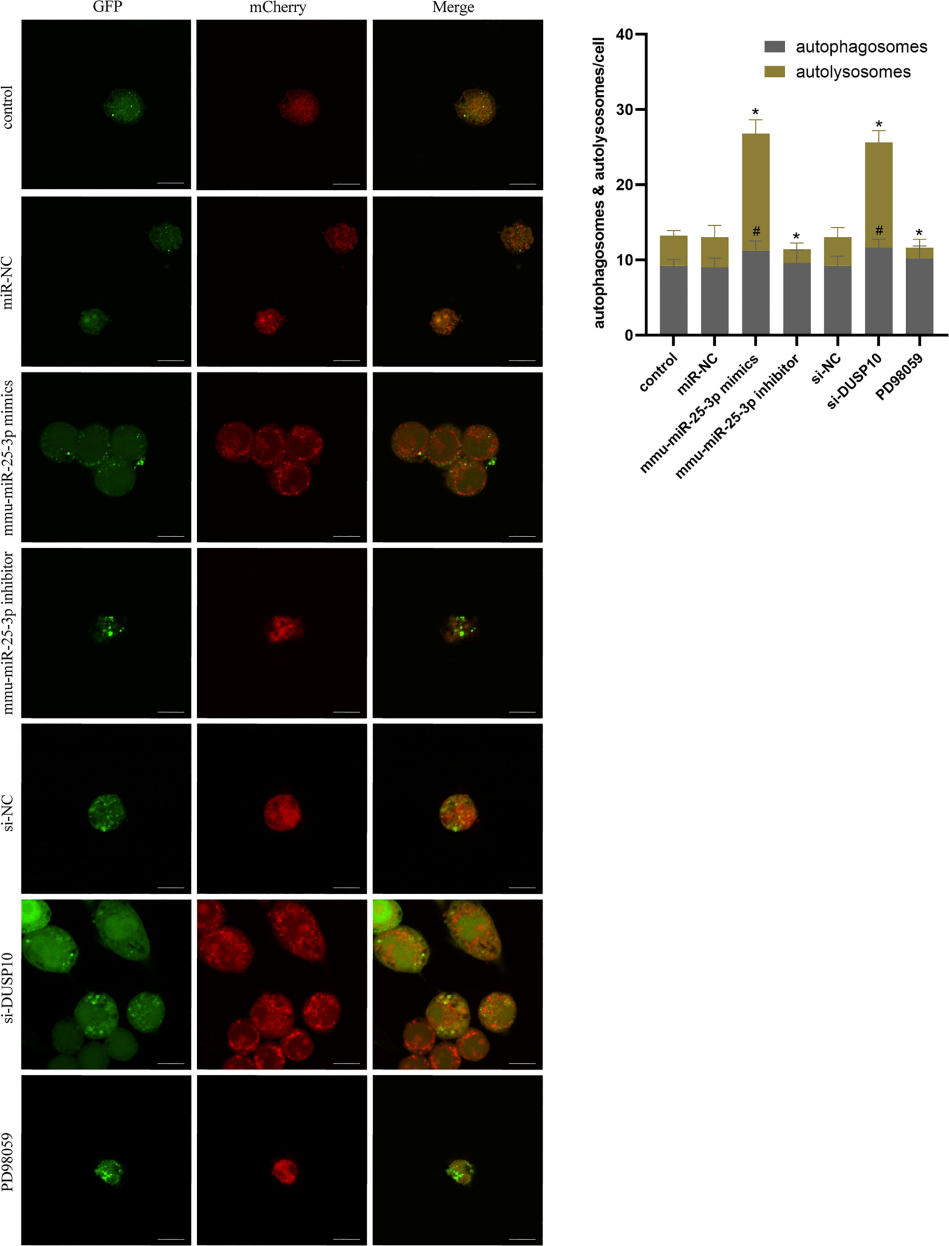
Autophagy flux analysis in each group. RAW264.7 cells were infected with pAAV-mCherry-GFP-LC3B adenovirus (MOI = 100) for 24 h prior to 8 h of BCG infection (MOI = 10). mCherry-LC3B and GFP-LC3B puncta were observed by confocal microscopy in the control group (only infected with BCG, no other interventions) and each intervention group (miR-NC, mmu-miR-25-3p mimics, mmu-miR-25-3p inhibitor, si-DUSP10, si-NC, and PD98059). All the experiments were repeated 3 times independently. Scale bars: 10 μm. #p < 0.05 (compared with autophagosomes of control), *p < 0.05 (compared with autolysosomes of control).

**Figure 9:**
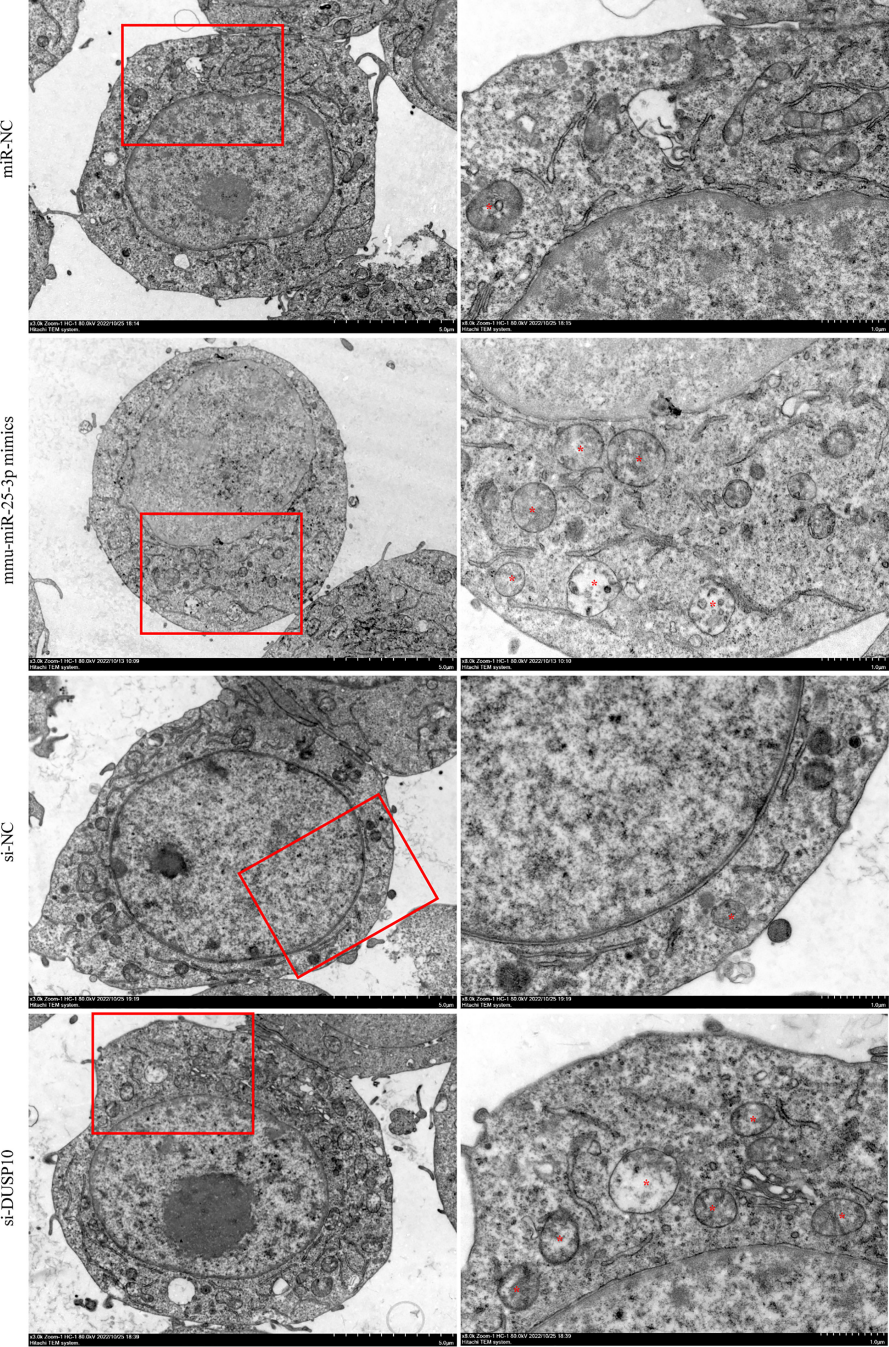
Observation of autophagic vesicles. After 8 h of BCG infection at MOI 10, autophagic vesicles (marked with red *) were observed by TEM in miR-NC, mmu-miR-25-3p mimics, si-NC, and si-DUSP10 groups. All the experiments were repeated 3 times independently. Scale bars: 5 μm (×3.0k), 1μm (×8.0k).

Since phosphorylation activates the MAPK signaling pathway, dephosphorylation of the members of the DUSP family plays a key role in controlling MAPK signaling (Huang et al., 2019; Ye et al., 2019). Also, DUSP10 suppresses the activation of ERK (Nomura et al., 2012). Next, we investigated whether the ERK1/2 signaling was regulated by mmu-miR-25-3p/DUSP10 in BCG-infected RAW264.7 macrophages. qRT-PCR showed that the overexpression of mmu-miR-25-3p and knockout of DUSP10 by siRNA significantly downregulated the expression level of *DUSP10* mRNA (p < 0.05) (**Figure 7A**). Western blotting assay confirmed that both the overexpression of miR-25-3p and knockdown of DUSP10 downregulate the level of DUSP10 (p < 0.05) and upregulate the level of p-ERK1/2 (p < 0.05); this trend was reversed by inhibiting the expression of miR-25-3p, the expression trend was reversed, that is, the expression level of DUSP10 was upregulated (p < 0.05) and the expression level of p-ERK1/2 was down-regulated (p < 0.05) (**Figure 7B**). Immunoelectron microscopy assay further demonstrated a decreased abundance of DUSP10 in BCG-infected cells with overexpression of miR-25-3p and knockdown of DUSP10 and an increased abundance of DUSP10 in BCG-infected cells by inhibiting miR-25-3p (**Figure 7C**). Next, we further the expression levels of LC3-II, beclin1, Atg5, and Atg7, and found that overexpressing mmu-miR-25-3p and knocking down the expression of DUSP10 significantly upregulates the levels of the above four autophagy-related proteins (p < 0.05) (**Figure 7D**). After inhibiting mmu-miR-25-3p and using ERK1/2 inhibitor PD98059, the expression levels of the above four proteins were significantly downregulated (p < 0.05) (**Figure 7D**). The mmu-miR-25-3p/DUSP10-mediated autophagy was further corroborated by ascertaining autophagy flux in cells expressing pAAV-mCherry-GFP-LC3B transduced by adenovirus. The autophagosome-lysosome fusion, as determined by quantity of red puncta in BCG-infected cells. Obviously, the autophagy activities were enhanced significantly (more quantity of yellow and red puncta) in BCG-infected cells with overexpressing mmu-miR-25-3p or knocked down the level of DUSP10 (p < 0.05), while the autophagy activities were significantly reduced (less quantity of red puncta) in BCG-infected cells by inhibiting mmu-miR-25-3p or using ERK1/2 inhibitor PD98059 (p < 0.05) (**Figure 8**). TEM further confirmed that the number of autophagosomes in BCG-infected cells with overexpressing mmu-miR-25-3p or knocked down DUSP10 was significantly higher than that in BCG-infected cells *via* miR-NC and si-NC (**Figure 9**).

### 3.6 mmu-miR-25-3p/DUSP10/ERK1/2 regulates BCG survival in Raw264.7 macrophages

**Figure 10:**
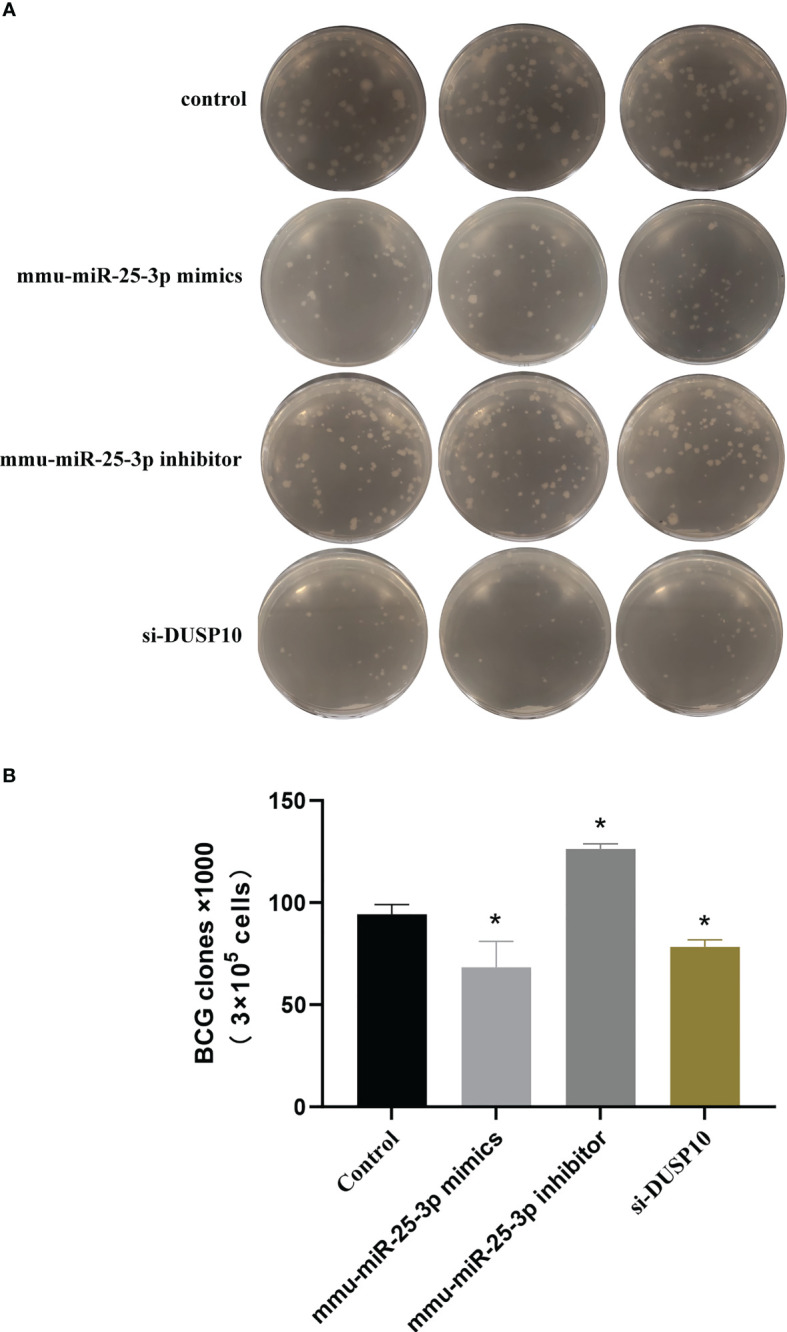
Results of mycobacteria CFU determination. **(A)** Number of mycobacteria CFUs in different groups after ×10^6^ dilution. **(B)** CFU comparison in control, mmu-miR-25-3p mimics group, mmu-miR-25-3p inhibitor, and si-DUSP10 groups. All the experiments were repeated 3 times independently. *p < 0.05.

The CFU assay results showed that compared to the control group (BCG-infected cells alone) the number of CFUs decreased significantly in the mmu- miR-25-3p mimics and si-DUSP10 groups (p < 0.05) and increased markedly in the mmu-miR-25-3p inhibitor group (p < 0.05) (**Figures 10A, B** **)**.

## 4 Discussion

In the present study, enrichment analysis of 27 differentially expressed miRNAs (**Table 1**) screened from exosomes of Raw264.7 macrophages infected with BCG in our previous study (Zhan et al., 2022) revealed that their target genes were closely related to autophagy regulation. The results of GO enrichment analysis showed that most of the target gene products were located in the cell membrane, cytoplasm, plasma membrane, nucleus, and mitochondria. Their molecular functions are mainly concentrated in protein binding with metal ions, G protein-coupled receptor activity, and transferase activity. KEGG enrichment analysis showed that these target genes were widely involved in autophagy and PI3K-Akt, mTOR, Rap1, Ras, and MAPK signaling pathways. Similar findings were previously reported by Aplipoor et al. (Alipoor et al., 2017), who identified a panel of exosomal miRNAs after stimulation of human monocyte-derived macrophages, which are involved in host metabolic processes and cell signal transduction, using BCG. As mentioned in the results section, considering the number of target genes enriched by each item and their rich factors, we selected MAPK signaling pathway, autophagy-animal, and mTOR signaling pathway as the focus of further analysis. Herein, we used Metascape to conduct an in-depth data mining and online analysis of functional visualization of these target genes, and the results showed that autophagy and PI3K-Akt, mTOR, Ras, MAPK, and JAK-STAT signaling pathways are top-ranked related signaling pathways. Although the ranking of autophagy is not the top, but it has been proved that autophagy is closely related to the survival of MTB retained in macrophages (Gutierrez et al., 2004; Amano et al., 2006). Considering the above results comprehensively, we finally chose MAPK signaling pathway and autophagy as the direction of further analysis. 6/27 miRNAs are highly expressed, and among them, mmu-miR-25-3p has the highest fold change (1.33) (**Table 1**); hence, it was selected to further analyze the Hub genes of mmu-miR-25-3p.

Autophagy is a cellular “self-feeding” process that is the innate and adaptive immune defense of host cells against intracellular pathogens (Bah and Vergne, 2017). Based on the concept of autophagy-mediated bacterial clearance, signal transduction pathways involved in the autophagy process may be potential new targets for anti-tuberculosis therapy (Dara et al., 2019). It has also been confirmed that MAPK signaling pathway is involved in the regulation of autophagy (Moruno-Manchon et al., 2013; Zhou et al., 2015). Therefore, we analyzed target genes of mmu-miR-25-3p related to MAPK signaling pathway and autophagy through CytoHubba. Finally, *Dusp10*, *Akt1*, *Mapk8*, *Rps6ka4*, *Elk4*, *Map2k4*, *Tsc1*, *Pik3ca*, *Prkaa2*, *Pik3r3*, and *Pik3cb* were identified as hub genes. Since phosphorylation is required to activate MAPK signal transduction pathways, dephosphorylation of DUSP (bispecial protein phosphatase) family members plays a key role in controlling MAPK signaling. Wang et al. (Wang et al., 2016) found that DUSP1 inhibited autophagy of ovarian cancer cells by negatively regulating MAPK/ERK pathway, and inhibition of DUSP1 could enhance rapamycin-induced autophagy. Zhou et al. (Zhao et al., 2020) confirmed that DUSP10 (also known as MKP-5) alleviates lipid toxin-induced islet cell dysfunction and apoptosis by inhibiting autophagy. Nomura et al. (Nomura et al., 2012) observed that DUSP10 suppresses the activation of ERK.

Therefore, we hypothesized that mmu-miR-25-3p regulates macrophage autophagy by targeting DUSP10, and that this regulation affects the outcome of MTB infection. Mmu-miR-25-3p is seems to be a novel immunoregulatory target for effective control of *Mycobacterium* infection, and may be crucial in guiding the development of exosome drug delivery systems loaded with specific regulators simultaneously. The prediction of TargetScan (Mouse v8.0) and the validation of the dual luciferase reporter gene assay results demonstrated that the seed sequence of mmu-miR-25-3p targeted binds with the 3’-UTR of DUSP10, and that *DUSP10* is a target gene of mmu-miR-25-3p. Finally, the regulatory mechanism of mmu-miR-25-3p in BCG-induced macrophage autophagy *via* DUSP10 expression and its effect on the survival of intracellular BCG was verified *in vitro*.

Another study confirmed that DUSP10 promotes the transformation of macrophages from M1 to M2 (Lu et al., 2020). The M1-type macrophages participate in the positive immune response and carry out immune surveillance by secreting pro-inflammatory cytokines and chemokines and presenting antigens exclusively. Using murine RAW264.7 macrophage cells *in vitro*, the current results demonstrated that the expression of *DUSP10* mRNA was dynamically decreased within 0–12 h after BCG infection, while the protein level was continuously and dynamically decreased after BCG infection. Contrary to the expression trend of *DUSP10* mRNA, the expression level of mmu-miR-25-3p increased dynamically within 0–8 h after BCG infection, i.e., reached a peak at 4 h and returned to the baseline level at 8 h post-BCG infection. miRNAs downregulate the expression of their target genes through two post-transcriptional mechanisms: mRNA cleavage or translation inhibition (Bartel, 2004). Therefore, the downregulated expression of *DUSP10* mRNA in this study may be related to the regulatory effect of mmu-miR-25-3p through mRNA cleavage. In addition, we found that the expression level of LC3-II in macrophages increased dynamically after BCG infection and reached a peak at 8 h after infection. Considering that after BCG infection, cell viability decreases significantly with the passage of culture time, we considered that 8 h after BCG infection is the best time point to observe autophagy and conduct a further experimental intervention. To better understand the effect of mmu-miR-25-3p/DUSP10 on autophagy of macrophages after BCG infection, autophagic markers, Beclin1 (Liang et al., 1999), Atg5, and Atg7(Romanov et al., 2012) were further examined for autophagosome membrane formation and extension, respectively. At 8 h after BCG infection, the levels of Beclin-1, Atg7, and Atg5 were significantly higher than those of uninfected macrophages, indicating that BCG induces autophagy in macrophages. Both autophagy flux (pAAV-mCherry-GFP-LC3B adenovirus) and transmission electron microscopy assays further confirmed that BCG infection induces autophagy in macrophages. This finding is consistent with the study of Luo et al. (Luo et al., 2020), which demonstrated that the autophagy of macrophages was enhanced after BCG infection.

Since phosphorylation is required to activate the MAPK signaling pathway, dephosphorylation of the members of the DUSP family plays a key role in controlling MAPK signaling(Huang et al., 2019; Ye et al., 2019). DUSP10 is shown to suppress the activation of ERK (Nomura et al., 2012; Ng et al., 2017). Next, we investigated whether ERK1/2 signaling was regulated by mmu-miR-25-3p/DUSP10 in BCG-infected RAW264.7 macrophages. Interestingly, a decreased expression of DUSP10 was detected in BCG-infected RAW264.7 cells with mmu-miR-25-3p mimics and DUSP10 knockdown (si-DUSP10). However, using mmu-miR-25-3p inhibitor exhibited the opposite effect in BCG-infected RAW264.7 cells. Conversely, an enhanced expression of p-ERK1/2, Beclin1, Atg5, and Atg7 was detected in BCG-infected RAW264.7 cells with mmu-miR-25-3p mimics and DUSP10 knockdown (si-DUSP10) and a decreased expression of p-ERK1/2, Beclin1, Atg5, and Atg7 was detected in BCG-infected RAW264.7 cells with mmu-miR-25-3p inhibitor. Park et al. (Park et al., 2014) confirmed the activation of ERK pathway in bare−FeNPs−induced autophagy in RAW264.7 and observed upregulated levels of Beclin1, Atg5 and LC3B. This finding is consistent with that of our study. Therefore, we concluded that mmu-miR-25-3p promotes the phosphorylation of ERK1/2 by inhibiting DUSP10, thus promoting the initiation of autophagy to enhance BCG-induced autophagy in RAW264.7 cells. Further autophagy flux assay confirmed that overexpression of mmu-miR-25-3p and downregulation of DUSP10 expression by si-DUSP10 can increase the number of autophagosome and autolysosomes in macrophages, which indicated that overexpression of mmu-miR-25-3p or DUSP10 knockdown can enhance the autophagy activity of macrophages. And TEM confirmed that overexpression of mmu-miR-25-3p or DUSP10 knockdown promotes the formation of autophagosomes in Raw264.7 cells infected with BCG.

Current studies have demonstrated that autophagy is closely related to the survival of mycobacteria in macrophages, and induction of autophagy can effectively eliminate the intracellular residual mycobacteria (Gutierrez et al., 2004; Amano et al., 2006; Singh et al., 2006; Biswas et al., 2008). Finally, the CFU was determined to estimate the intracellular bacterial load and mycobacteria clearance rate. The results showed that mmu-miR-25-3p and DUSP10 are closely related to the bacterial load in Raw264.7 cells. By inhibiting the expression of DUSP10, mmu-miR-25-3p reduces the bacterial load and survival rate of Raw264.7 cells.

In conclusion, mmu-miR-25-3p promotes the phosphorylation of ERK1/2 by inhibiting the expression of DUSP10. This enhances the BCG-induced autophagy of macrophages, which in turn reduces the bacterial load of intracellular *Mycobacterium* and facilitates the clearance of residual mycobacteria. mmu-miR-25-3p has significant potential as a target for anti-tuberculosis immunotherapy and can be the optimal miRNA loaded into exosomal drug delivery system in our future studies.

## Data availability statement

The datasets presented in this study can be found in online repositories. The names of the repository/repositories and accession number(s) can be found below: https://ngdc.cncb.ac.cn/gsa/,CRA006010.

## Author contributions

ZG: conceptualization and writing - review & editing. RM: methodology and resources. WY: investigation, formal analysis, writing - original draft. XZ: investigation, software, visualization. WL: data curation. YZ: data curation. GX: supervision.
